# Duration-Dependent Ketone Body Responses to Fasting: A Systematic Review, Meta-Analysis, and Meta-Regression

**DOI:** 10.3390/ijms27219570

**Published:** 2026-10-26

**Authors:** Süleyman Ulupınar, Merve Terzi, Nedim Malkoç, Ayşe Çamli, İzzet İnce, Cebrail Gençoğlu, Serhat Özbay

**Affiliations:** 1 Faculty of Sports Sciences, Erzurum Technical University, 25100 Erzurum, Turkey; suleyman.ulupinar@erzurum.edu.tr (S.U.); cebrail.gencoglu@erzurum.edu.tr (C.G.); serhat.ozbay@erzurum.edu.tr (S.Ö.); 2 Department of Nutrition and Dietetics, Faculty of Health Sciences, Istanbul Yeni Yuzyil University, 34010 Istanbul, Turkey; 3 Department of Exercise and Sport Sciences, Hamidiye Faculty of Life Sciences, University of Health Sciences, 34100 Istanbul, Turkey; nedim.malkoc@sbu.edu.tr; 4 Department of Nutrition and Dietetics, Faculty of Health Sciences, Erzurum Technical University, 25000 Erzurum, Turkey; 5 Faculty of Sports Sciences, Ankara Yıldırım Beyazıt University, 06000 Ankara, Turkey; izzetince@aybu.edu.tr

**Keywords:** water-only fasting, ketone bodies, β-hydroxybutyrate, acetoacetate, duration-dependent effects, meta-analysis, meta-regression

## Abstract

Prolonged fasting with no caloric intake induces a metabolic shift characterized by increased fat oxidation and ketone production. However, the magnitude of circulating ketone responses and their dependence on fasting duration have not been quantitatively synthesized. This systematic review and meta-analysis evaluated the effects of water-only fasting and other prolonged fasting protocols involving complete caloric abstinence on circulating ketone biomarkers and examined whether fasting duration moderates these responses. PubMed, Web of Science, and Scopus were searched for human studies published between 2000 and 2025. Eligible studies reported pre–post measurements of β-hydroxybutyrate (BHB), acetoacetate, or total plasma ketones following water-only or other non-caloric fasting protocols. Random-effects meta-analyses were performed using Hedges’ g. Duration effects were assessed through subgroup analyses (≤3 days vs. >3 days) and random-effects meta-regression with study-level cluster-robust standard errors. Thirty studies providing 44 effect sizes were included. Fasting significantly increased BHB (g = 2.794), total plasma ketones (g = 3.260), and acetoacetate (g = 1.731). Greater elevations in BHB and total plasma ketones were observed with fasting durations exceeding three days. Meta-regression indicated significant positive associations between fasting duration and responses for BHB and total plasma ketones, whereas no significant association was detected for acetoacetate. Despite substantial heterogeneity for BHB and total plasma ketones, sensitivity analyses supported the robustness of the pooled effects. Small-study effects were detected for BHB, whereas funnel-plot asymmetry for total plasma ketones was considered exploratory because of the limited number of studies. Water-only fasting and other prolonged fasting protocols involving complete caloric abstinence are associated with marked increases in circulating ketone bodies, with a clear duration-dependent pattern observed particularly for BHB and total plasma ketones.

## 1. Introduction

Fasting represents a fundamental metabolic challenge that has shaped human physiology through evolutionary pressures, requiring coordinated adaptations to maintain energy homeostasis during periods of food deprivation [1,2]. In the absence of exogenous nutrient supply, the organism undergoes a progressive shift in substrate utilization, transitioning from glycogen-dependent glucose metabolism toward increased reliance on fatty acid oxidation and alternative energy sources [3,4,5]. This adaptive process, often described as a metabolic switch, enables the preservation of vital organ function while minimizing protein catabolism and maintaining systemic energy balance [6,7]. In recent years, fasting-based interventions—including prolonged fasting protocols involving complete caloric abstinence—have attracted renewed scientific and clinical interest due to their potential effects on metabolic health, body composition, and cellular resilience [1,8,9]. However, despite growing applications across diverse contexts, the underlying physiological responses to fasting remain incompletely characterized, particularly with respect to the temporal dynamics of key metabolic pathways.

A central component of this metabolic adaptation is hepatic ketogenesis, whereby fatty acids are converted into ketone bodies—primarily β-hydroxybutyrate (BHB), acetoacetate, and acetone—which serve as alternative oxidative fuels for peripheral tissues and the central nervous system [10,11,12]. As hepatic glycogen stores become depleted, typically within the first 24 h of fasting, lipolysis and fatty acid oxidation accelerate, leading to a progressive rise in circulating ketone concentrations [4,5,13]. Over subsequent days, ketone bodies increasingly contribute to whole-body energy metabolism, with BHB emerging as the dominant circulating ketone due to its greater stability and transport efficiency [14,15,16]. This transition reflects a coordinated metabolic shift designed to spare glucose, reduce proteolysis, and sustain cerebral energy demands during prolonged caloric deprivation [13,17,18]. In this context, β-hydroxybutyrate represents the primary circulating ketone body, acetoacetate reflects hepatic ketogenesis, and total plasma ketones provide an integrated measure of systemic ketone availability, supporting their combined use as complementary biomarkers of fasting-induced metabolic adaptation. Importantly, ketone accumulation during fasting is time dependent and may involve distinct metabolic phases rather than a strictly linear physiological trajectory from the onset of fasting. Relatively modest elevations are generally observed during early fasting, followed by larger increases as fasting continues [19,20]. Accordingly, fasting duration is likely to be an important moderator of circulating ketone responses, although the precise shape and magnitude of this relationship remain incompletely characterized. However, although this temporal pattern is well recognized at the physiological level, its magnitude, consistency, and quantitative characteristics across human studies have not been systematically established.

Despite extensive investigation of fasting-induced metabolic changes, existing human studies examining circulating ketone responses remain fragmented and methodologically heterogeneous [16,21,22,23]. Sample sizes are frequently small [6,24,25], fasting durations vary widely—from short-term (24–36 h) to extended protocols lasting up to 10 days [23,26]—and outcome reporting differs across studies with respect to specific ketone biomarkers and measurement time points [14,16,27]. Consequently, although individual investigations consistently report elevations in circulating ketone bodies during fasting, the field lacks a quantitative synthesis that can integrate these findings into a unified framework. To date, no meta-analytic study has systematically quantified the magnitude of fasting-induced ketone responses across different durations, nor compared the relative effect sizes between early and prolonged fasting phases. In particular, it remains unclear whether the transition beyond the initial 48–72 h represents a gradual increase or a disproportionately amplified metabolic response, and no study has formally modeled this relationship using continuous meta-regression approaches. Therefore, the present systematic review and meta-analysis aimed to evaluate the duration-dependent effects of prolonged fasting without caloric intake on circulating ketone biomarkers in adults.

## 2. Methods

### 2.1. Literature Search Strategy

A comprehensive systematic literature search was conducted in PubMed, Web of Science (WoS), and Scopus to identify human studies examining the duration-dependent effects of fasting on circulating ketone bodies. The search covered publications from January 2000 through December 2025 and was restricted to peer-reviewed English-language articles.

In PubMed, the following search strategy was applied: (fasting[Title/Abstract] OR “prolonged fasting”[Title/Abstract] OR “water-only fasting”[Title/Abstract] OR “water fasting”[Title/Abstract] OR starvation[Title/Abstract] OR “caloric restriction”[Title/Abstract]) AND (“ketone bodies”[MeSH Terms] OR “beta-hydroxybutyrate”[Title/Abstract] OR β-hydroxybutyrate[Title/Abstract] OR BHB[Title/Abstract] OR “plasma ketones”[Title/Abstract] OR acetoacetate[Title/Abstract]) AND (duration[Title/Abstract] OR time-dependent[Title/Abstract] OR days[Title/Abstract]) AND Humans[MeSH Terms], yielding 104 records.

Web of Science was searched using the following Boolean query: ((fasting OR “prolonged fasting” OR “water-only fasting” OR “water fasting” OR starvation OR “caloric restriction”) AND (“ketone bodies” OR “beta-hydroxybutyrate” OR “β-hydroxybutyrate” OR BHB OR “plasma ketones” OR acetoacetate) AND (duration OR “time-dependent” OR days)), which retrieved 615 records.

Scopus was queried using a TITLE–ABS–KEY strategy: TITLE-ABS-KEY ((fasting OR “prolonged fasting” OR “water-only fasting” OR “water fasting” OR starvation OR “caloric restriction”) AND (“ketone bodies” OR “beta-hydroxybutyrate” OR “β-hydroxybutyrate” OR BHB OR “plasma ketones” OR acetoacetate) AND (duration OR “time-dependent” OR days)) AND PUBYEAR > 1999 AND PUBYEAR < 2027 AND (LIMIT-TO (LANGUAGE, “English”)), yielding 439 records.

**Figure 1:**
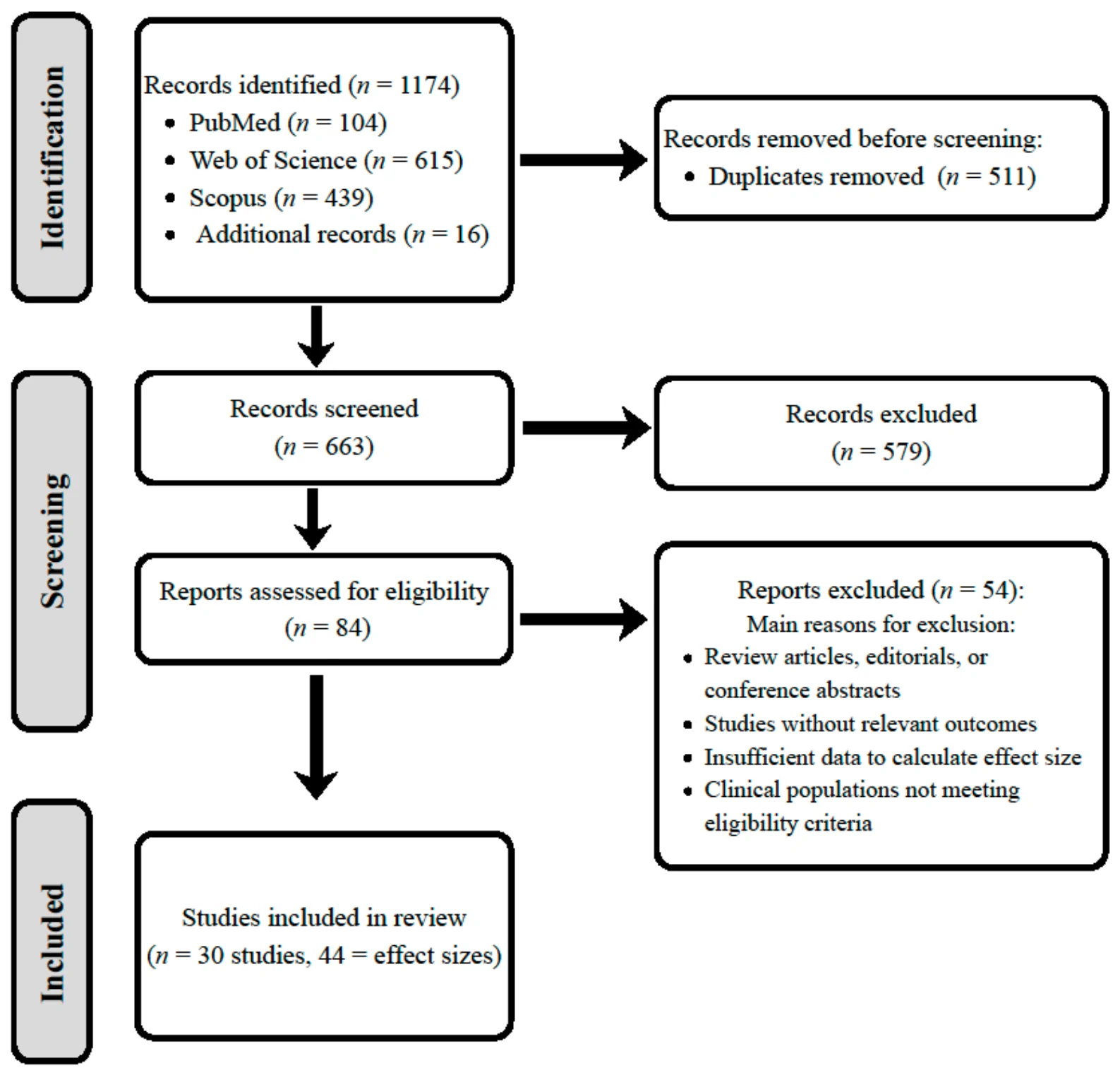
PRISMA flow diagram.

The study identification and selection process followed PRISMA 2020 guidelines [28], and a PRISMA flow diagram was constructed to document each stage of screening and eligibility assessment (Figure 1).

### 2.2. Eligibility Criteria

Studies were considered eligible if they met the following criteria: (i) inclusion of human participants aged ≥18 years, irrespective of sex or ethnicity; healthy adults, mixed populations, and clinical populations were eligible when the fasting protocol, outcome assessment, and available pre–post data were considered sufficiently comparable with the broader eligible literature; (ii) implementation of a fasting protocol involving complete abstinence from caloric intake, with a minimum duration of 24 h; water, mineralized water, and non-caloric beverages such as unsweetened black coffee or tea were permitted, provided that no energy-containing foods, beverages, or supplements were consumed during the fasting period; (iii) experimental, quasi-experimental, or longitudinal pre–post study designs reporting baseline and post-fasting measurements; (iv) assessment of at least one circulating ketone biomarker, including β-hydroxybutyrate, acetoacetate, or total plasma ketones; and (v) availability of sufficient quantitative data to permit effect size calculation, either directly (mean and standard deviation) or through established conversion methods from standard error, median and interquartile range, or digitized graphical data (WebPlotDigitizer, version 4.x). Only full-text, peer-reviewed articles published in English between January 2000 and 31 December 2025 were included.

Studies were excluded if they involved animal or in vitro experiments; incorporated any caloric food, beverage, or energy-containing supplement during the fasting period; employed religious fasting practices (e.g., Ramadan) or intermittent fasting regimens (e.g., time-restricted feeding or alternate-day fasting); involved clinical populations for which disease-related characteristics, treatment context, or protocol-specific factors were considered to substantially limit comparability with the broader fasting literature; or were non-original publications such as reviews, meta-analyses, conference abstracts, case reports, or editorials lacking primary quantitative data.

### 2.3. Data Extraction and Transformation

Two independent reviewers (A.Ç. and S.U.) extracted data from all eligible studies using a standardized data extraction form. Extracted variables included study identifiers (author, year, country), participant characteristics (sample size, sex, age), fasting duration, study design, fasting protocol details, and circulating ketone outcomes (β-hydroxybutyrate, acetoacetate, or total plasma ketones). Discrepancies between reviewers were resolved through discussion until consensus was reached. When available, mean values and standard deviations (SDs) of pre- and post-fasting ketone concentrations were recorded directly. However, given the substantial heterogeneity in data reporting formats across studies, several transformation procedures were applied to ensure statistical comparability across datasets. To ensure consistency and prevent scaling errors during data handling, ketone concentration units were harmonized where necessary; however, as effect sizes were calculated using standardized mean differences, unit differences did not influence the magnitude of pooled estimates.

***Standard Error (SE) to Standard Deviation (SD):*** When results were expressed as mean ± SE, the SD was computed using:

$$SD=SE\;\times \;\sqrt{n}$$

***Median and Interquartile Range (IQR) to Mean and SD:*** For studies reporting medians and IQRs, the mean and SD were estimated using established transformation equations proposed by Hozo et al. and refined by Wan et al. [29,30]. The Wan et al. approach was applied using the sample-size-adjusted divisor, with the appropriate transformation selected according to the available summary statistics and sample size.

***Graphically Reported Data:*** For studies presenting data only in graphical form (e.g., bar or line charts), quantitative values were extracted using WebPlotDigitizer (version 4.x) [31]. Each graph was digitized twice by separate reviewers to minimize measurement error, and the averaged values were used in subsequent analyses.

***Fasting Duration Standardization:*** Fasting duration was harmonized across studies by converting all reported units (hours, days, or ranges) into a single continuous variable expressed in days. For range values (e.g., “3–5 days”), the midpoint was used. Independent parallel groups undergoing different fasting durations were retained as separate effect-size estimates because they represented distinct participant samples. In contrast, when the same participants contributed measurements at multiple fasting durations, the longest available fasting duration was retained for the primary overall meta-analysis to avoid treating repeated observations from the same individuals as independent. For subgroup analyses, duration-specific measurements were retained when they contributed to different predefined fasting-duration categories (≤3 days vs. >3 days); when multiple measurements from the same participants fell within the same category, the longest eligible duration was retained. Because such repeated measurements could contribute to both duration categories, subgroup comparisons involving these data were interpreted as exploratory. Duration-specific repeated observations were retained for the meta-regression analysis, where their within-study clustering was explicitly accounted for using study-level cluster-robust standard errors.

### 2.4. Effect Size Calculation

The effect size for each study was expressed as Hedges’ g, representing the bias-corrected standardized mean difference between post-fasting and pre-fasting circulating ketone concentrations. For pre–post designs, the standardized mean difference was calculated using the standard deviation of the change score, thereby incorporating the dependency between repeated measurements; Hedges’ small-sample correction was then applied.

For each study, Hedges’ g was calculated as:

$$g=J\;\times \frac{M_{post}\;-\;M_{pre}\;}{{SD}_{change}}$$

where *M_post_* and *M_pre_* denote the mean ketone concentrations after and before fasting, respectively, and *J* denotes the small-sample bias-correction factor.

The standard deviation of the pre–post change scores was computed as:

$${SD}_{change}=\sqrt{{{SD}_{pre}}^{2}+\;{{SD}_{post}}^{2}-2r\cdot \left({SD}_{pre}\right)\cdot ({SD}_{post})}$$

where r represents the within-subject pre–post correlation. Because most studies did not report this correlation, the primary analyses assumed r = 0.5. To assess robustness to this assumption, sensitivity analyses were additionally conducted using r = 0.3 and r = 0.7 [32,33].

Independent parallel fasting groups comprising different participants were retained as separate effect-size estimates, including groups exposed to different fasting durations. By contrast, when the same participants contributed ketone measurements at multiple fasting durations, these repeated observations were not pooled as if they were independent. For the primary overall meta-analysis, the longest eligible fasting duration was retained for each repeated-measures study–biomarker combination. For duration subgroup analyses, repeated measurements were permitted to contribute separately when they fell into different predefined duration categories (≤3 days vs. >3 days), while only the longest eligible measurement was retained when multiple observations from the same participants occurred within the same category.

Accordingly, inverse-variance pooling of multiple within-study fasting durations or repeated time points was not used in the revised primary analyses. This approach was adopted to reduce artificial precision arising from correlated observations while preserving duration-specific information for analyses explicitly modeling fasting duration.

### 2.5. Statistical Analysis

All statistical analyses were conducted according to the methodological recommendations for the quantitative synthesis of pre–post intervention studies. Each ketone biomarker—including β-hydroxybutyrate, acetoacetate, and total plasma ketones—was analyzed separately.

#### 2.5.1. Overall Meta-Analysis

The pooled effect size for each biomarker was calculated using a random-effects model based on the DerSimonian–Laird (DL) method [34,35]. The random-effects framework accounts for both within-study sampling variability and between-study heterogeneity. The DL estimator was used for the primary analysis to maintain consistency across biomarkers and analyses. To evaluate the robustness of between-study variance estimation, additional random-effects models were fitted using restricted maximum likelihood (REML) and Paule–Mandel (PM) estimators [36]. Consistency of pooled estimates across alternative τ^2^ estimators was examined as part of the sensitivity analyses.

Study-specific Hedges’ g estimates were combined using inverse-variance weighting under the random-effects framework, with weights incorporating both within-study sampling variance and estimated between-study variance.

Between-study heterogeneity was quantified using Cochran’s Q statistic, I^2^, and τ^2^. The number of independent study-level estimates contributing to each biomarker-specific analysis was used when calculating heterogeneity statistics. Values of I^2^ around 25%, 50%, and 75% were interpreted as low, moderate, and high heterogeneity, respectively.

In addition to conventional 95% confidence intervals, Hartung–Knapp confidence intervals and 95% prediction intervals were calculated to provide complementary assessments of uncertainty around the pooled effect and the range of effects that might be expected in comparable future studies.

Sensitivity analyses were performed to confirm the stability of pooled estimates across alternative τ^2^ estimators and correlation assumptions [37]. These analyses included alternative pre–post correlation values (r = 0.3 and r = 0.7) and, where relevant, an analysis restricted to the largest sample within potentially overlapping study cohorts.

In addition to the primary analyses, the consistency of effects was qualitatively assessed by examining the direction and distribution of individual study estimates in the forest plots.

#### 2.5.2. Subgroup Analysis

To examine whether fasting duration moderated ketone responses, studies were stratified into two predefined duration categories (≤3 days vs. >3 days), guided by established physiological evidence indicating that hepatic glycogen depletion and the metabolic transition toward predominant ketone production typically occur within approximately 48–72 h of fasting [13,38]. The 3-day boundary was used as a physiologically informed and analytically pragmatic classification rather than as a strict biological threshold. All fasting protocols lasting exactly 72 h were consistently classified in the ≤3-day subgroup.

Each subgroup was analyzed separately using random-effects models, and between-group heterogeneity was evaluated using the *Q*_Between_ statistic (χ^2^ distribution). A statistically significant *Q*_Between_ value (*p* < 0.05) was interpreted as evidence that effect sizes differed between the two duration categories. This binary classification was implemented to ensure adequate subgroup sizes and to reduce sparse-data issues associated with multi-level stratification. Independent parallel groups exposed to different fasting durations were retained as separate estimates. For repeated-measures studies, duration-specific observations were retained when they fell into different predefined fasting-duration categories (≤3 days vs. >3 days); when more than one observation from the same participants fell within the same category, only the longest eligible duration was retained. Because repeated measurements from the same participants could therefore contribute to both duration categories, subgroup comparisons involving such data were regarded as exploratory and interpreted cautiously.

#### 2.5.3. Linear Meta-Regression Analysis

In addition to subgroup comparisons, meta-regression analyses were performed to examine the association between fasting duration (expressed as a continuous moderator in days) and changes in circulating ketone concentrations. Random-effects weighted least-squares (WLS) meta-regression models were fitted separately for each ketone biomarker. All eligible duration-specific estimates were retained in these models, including repeated fasting-duration observations contributed by the same study. To account for the resulting within-study dependence, statistical inference was based on study-level cluster-robust (sandwich) standard errors. Regression coefficients (*β*) were estimated to represent the expected change in standardized ketone response per additional day of fasting.

The moderator slope was evaluated using cluster-robust *t*-tests, with degrees of freedom based on the number of independent study clusters (*df* = *m* – 2\), where *m* denotes the number of contributing studies). Two-sided *p* < 0.05 was considered statistically significant. The proportion of between-study heterogeneity accounted for by fasting duration was additionally summarized from the reduction in *τ*^2^ relative to the corresponding intercept-only model. Residual heterogeneity was evaluated using *Q*_E_.

Meta-regression results were visualized by superimposing fitted regression lines on scatter plots of individual study effect sizes, weighted by inverse variance. Because the fitted model evaluates a linear statistical association, the estimated slope was interpreted as a summary of the duration–effect relationship rather than evidence that the underlying physiological ketone trajectory is itself strictly linear.

***Visualization and Reporting*:** Forest plots were used to display individual study effect sizes and pooled estimates with corresponding 95% confidence intervals for each ketone biomarker. Funnel plots were generated to visually assess potential asymmetry. Meta-regression plots displayed duration-specific effect-size estimates together with the fitted linear relationship. All figures were produced in black and white to ensure visual consistency across biomarkers.

#### 2.5.4. Statistical Software

All analyses were performed in Python (version 3.10) using custom-written scripts that utilized the pandas, numpy, scipy.stats, and matplotlib libraries. Random-effects modeling, subgroup comparisons, linear duration meta-regression with study-level cluster-robust standard errors, sensitivity analyses, heterogeneity estimation, and graphical analyses were implemented within this unified analytical pipeline in accordance with established meta-analytic principles.

### 2.6. Publication Bias Assessment

Potential publication bias was evaluated using both visual and statistical approaches. Funnel plots were generated for each ketone biomarker to examine the symmetry of effect size distributions relative to study precision (standard error). In the absence of small-study effects or other sources of asymmetry, studies are expected to form a broadly symmetrical inverted funnel around the pooled effect size. Formal statistical testing of funnel-plot asymmetry was restricted to biomarkers represented by at least 10 independent study-level estimates. Accordingly, Egger’s regression intercept test was performed only for β-hydroxybutyrate. Egger’s regression examines the association between standardized effect sizes and their precision, and a significant intercept (*p* < 0.05) was interpreted as evidence of funnel-plot asymmetry consistent with possible small-study effects, rather than as definitive evidence of publication bias. For plasma ketones and acetoacetate, the number of independent estimates was below 10; therefore, Egger’s test was not performed and funnel plots were assessed visually only. Any apparent asymmetry in these biomarkers was interpreted as exploratory because it may reflect small-study effects, selective publication, or genuine between-study heterogeneity.

### 2.7. Risk of Bias Considerations

Methodological quality was appraised using the National Institutes of Health (NIH) Quality Assessment Tool for Before–After (Pre–Post) Studies With No Control Group [39], which is specifically designed for the single-arm and within-subject pre–post designs that predominated among the included studies. Each study was rated against 12 items covering the clarity of the study objective; the specification of eligibility criteria; the representativeness of the participants; participant enrolment; sample-size adequacy; the description and consistency of the intervention; the definition, validity, reliability, and consistent assessment of outcomes; blinding of outcome assessors; loss to follow-up; the reporting of pre–post statistical tests; the use of repeated outcome measurements; and the appropriateness of group-level analysis. Item 8 (assessor blinding) was treated as not applicable because all outcomes were objective circulating-ketone concentrations measured by standardized biochemical assays or validated point-of-care analysers; item 12 was not applicable because all interventions were delivered at the individual rather than the group level. Two reviewers independently rated each study, and discrepancies were resolved through discussion and consensus. Item-level ratings and the corresponding justifications are provided in the Supplementary Materials (Table S1).

Each study was then assigned an overall rating of good, fair, or poor, integrating the NIH items with study-specific considerations including sample size, attrition, potential co-interventions, extraction of outcome data from figures, and probable participant overlap among cohorts. Borderline cases were rated conservatively but not punitively, recognising that all included studies were retrieved from major scientific databases and published in peer-reviewed journals. No study was excluded on the basis of this appraisal, as the eligibility criteria and selection process had already removed methodologically inappropriate or irrelevant reports.

Overall, 13 of the 30 included studies were rated good and 17 fair, with none rated poor. The most frequent limitations were small samples, the absence of a concurrent non-fasting comparator inherent to single-arm designs, incomplete reporting of participant characteristics, reliance on graphically extracted values in several reports, and probable participant overlap among a small number of prolonged-fasting cohorts—notably the 10-day fasting protocols reported by Yang et al. (2021) [21], Ma et al. (2021) [26], and Dai et al. (2022) [14], and the series of 8-day water-only fasting protocols conducted by a single research group (Ogłodek and Pilis (2021) [40], Letkiewicz et al. (2020) [41], Pilis et al. (2023) [42], Pilis et al. (2025) [43], and Stec et al. (2023) [44]). By contrast, risk of bias in outcome measurement was consistently low, as circulating ketone concentrations were quantified using standardized methods, and the within-subject (pre–post) structure of most studies provides an internal reference by comparing participants with their own baseline values, thereby reducing inter-individual variability. Accordingly, the pooled estimates should be interpreted as standardized within-person changes during fasting rather than definitive causal effects.

## 3. Results

### 3.1. Study Selection

A total of 1174 records were identified through database searches and additional sources (PubMed: n = 104; Web of Science: n = 615; Scopus: n = 439; additional records: n = 16). After removing 511 duplicate records, 663 records remained for title and abstract screening. Of these, 579 records were excluded, leaving 84 reports for full-text eligibility assessment. Subsequently, 54 reports were excluded for the following main reasons: non-original publications such as reviews, editorials, or conference abstracts; absence of relevant circulating ketone outcomes; insufficient quantitative data for effect-size calculation; or clinical populations not meeting the predefined eligibility criteria. Ultimately, 30 studies met the inclusion criteria and were included in the systematic review, yielding a total of 44 effect sizes for quantitative meta-analysis (Figure 1).

### 3.2. Study Characteristics

Detailed study characteristics, including fasting protocols, participant demographics, sample sizes, and outcome measures, are summarized in Table 1. Biomarker-specific outcomes comprised β-hydroxybutyrate (24 studies, 30 effect sizes), plasma ketones (6 studies, 11 effect sizes), and acetoacetate (3 studies, 3 effect sizes). Because three studies reported more than one ketone biomarker, these correspond to 30 unique studies contributing 44 effect sizes overall. For the overall meta-analyses, effect sizes were reduced to a single independent estimate per study, with the exception that the independent 2-day and 6-day fasting arms of one parallel-group trial were retained separately; this yielded 24, 7, and 3 study-level estimates for β-hydroxybutyrate, plasma ketones, and acetoacetate, respectively.

Fasting duration ranged from approximately 1 to 10 days. Among studies explicitly implementing water-only fasting, durations ranged from 24 h to approximately 10 days. Most protocols involved strict water-only fasting or complete caloric abstinence with calorie-free fluids, while a small number additionally permitted non-caloric beverages such as unsweetened black coffee or tea. Considerable methodological heterogeneity was observed across studies. Several investigations compared different fasting durations within the same cohort (e.g., 36 vs. 60 h; 2 vs. 6 days) or sampled the same participants repeatedly over the course of the fast, whereas others contrasted physiological responses under resting versus exercise conditions. Sex-specific analyses providing separate outcomes for women and men were reported in selected studies, and body composition–based comparisons contrasting lean and overweight/obese participants were conducted in several trials. Most studies used prospective intervention designs, including randomized crossover, parallel-group, or single-arm pre–post protocols, and study populations consisted predominantly of healthy adults spanning normal-weight to overweight/obese individuals, with per-study sample sizes ranging from 6 to 57 participants.

Ketone outcomes were obtained from plasma, serum, whole-blood, or capillary measurements using standardized biochemical assays or validated point-of-care analysers; where a study reported data only in graphical form, values were extracted by digitization (Section 2.3). Several prolonged-fasting cohorts showed probable participant overlap (Section 2.7), which was examined in a dedicated sensitivity analysis restricted to the largest sample within each overlapping cluster.

#### 3.2.1. Acetoacetate

##### Overall Meta-Analysis

**Figure 2:**
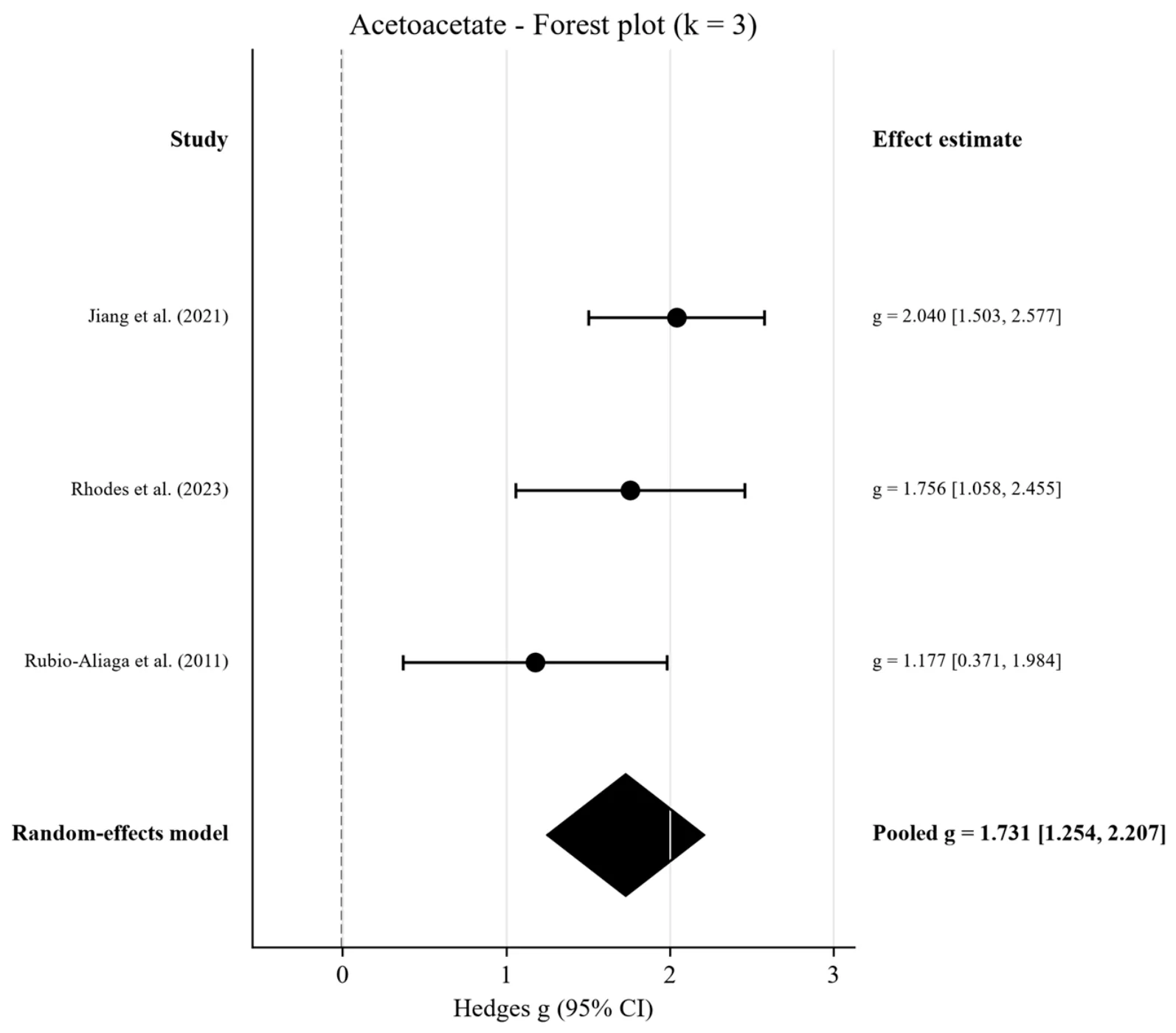
Forest plot of the effect of water-only fasting on circulating acetoacetate levels [11,12,49]. Circles indicate individual study effect estimates, while the rhombus represents the pooled effect estimate and its 95% confidence interval.

Three independent study-level estimates from three studies were included in the acetoacetate analysis (Figure 2). The random-effects model showed a large and statistically significant increase in circulating acetoacetate concentrations following water-only fasting (Hedges’ g = 1.731, SE = 0.243; 95% CI: 1.254 to 2.207; *p* < 0.001). Between-study heterogeneity was low to moderate (Q = 3.05, *p* = 0.218; I^2^ = 34.4%; τ^2^ = 0.062), indicating relatively limited variability in effect sizes across the three contributing studies.

##### Robustness and Sensitivity Analyses

The pooled effect remained statistically significant under Hartung–Knapp inference using the REML estimate of between-study variance (95% HK CI: 0.895 to 2.568). Estimates of τ^2^ were highly consistent across alternative estimators (DL = 0.062, PM = 0.061, REML = 0.059), indicating that the pooled result was not materially affected by the choice of heterogeneity estimator. The 95% prediction interval ranged from −2.639 to 6.100, indicating substantial uncertainty regarding the range of effects that might be observed in a future comparable study.

Sensitivity analyses using alternative assumptions for the pre–post correlation produced nearly identical pooled estimates (r = 0.3: g = 1.755, 95% CI: 1.289 to 2.221; r = 0.7: g = 1.706, 95% CI: 1.219 to 2.193). Analyses restricted to the longest fasting duration per study and those accounting for potentially overlapping cohorts yielded results identical to the primary analysis (g = 1.731, 95% CI: 1.254 to 2.207; k = 3). Thus, the direction and statistical significance of the pooled effect were unchanged across all sensitivity analyses.

##### Leave-One-Out Influence Analysis

Leave-one-out analyses showed that the pooled effect remained positive and statistically significant regardless of which individual study was excluded (Figure S1). Across the three iterations, the pooled estimate ranged from g = 1.503 when Jiang et al. (2021) [49] was omitted to g = 1.935 when Rubio-Aliaga et al. (2011) [12] was omitted. However, given that only three studies contributed to this outcome, the ability of the leave-one-out analysis to identify influential studies was inherently limited.

##### Subgroup Analysis by Fasting Duration

When studies were stratified according to fasting duration, fasting lasting ≤3 days was associated with a large and statistically significant increase in acetoacetate concentrations (k = 2; g = 1.503; 95% CI: 0.940 to 2.066). The single study involving fasting for >3 days also showed a large positive effect (k = 1; g = 2.040; 95% CI: 1.503 to 2.577). Because the >3-day subgroup contained only one study, a formal between-subgroup comparison was not considered statistically meaningful.

##### Duration Meta-Regression

An exploratory random-effects meta-regression was performed to examine fasting duration as a continuous moderator of the acetoacetate response. Using study-level cluster-robust standard errors, fasting duration was not significantly associated with effect size (β = 0.156 per day, cluster-robust SE = 0.100; t = 1.55; 95% CI: −1.119 to 1.430; *p* = 0.365). Given that the analysis was based on only three estimates from three independent studies, this result should be regarded as exploratory and interpreted with considerable caution.

##### Publication Bias

Egger’s regression test was not performed because fewer than 10 independent study-level estimates were available (k = 3). Visual inspection of the funnel plot did not indicate marked asymmetry (Figure S2); however, given the very small number of contributing studies, this assessment should be considered exploratory.

#### 3.2.2. β-Hydroxybutyrate (BHB)

##### Overall Meta-Analysis

**Figure 3:**
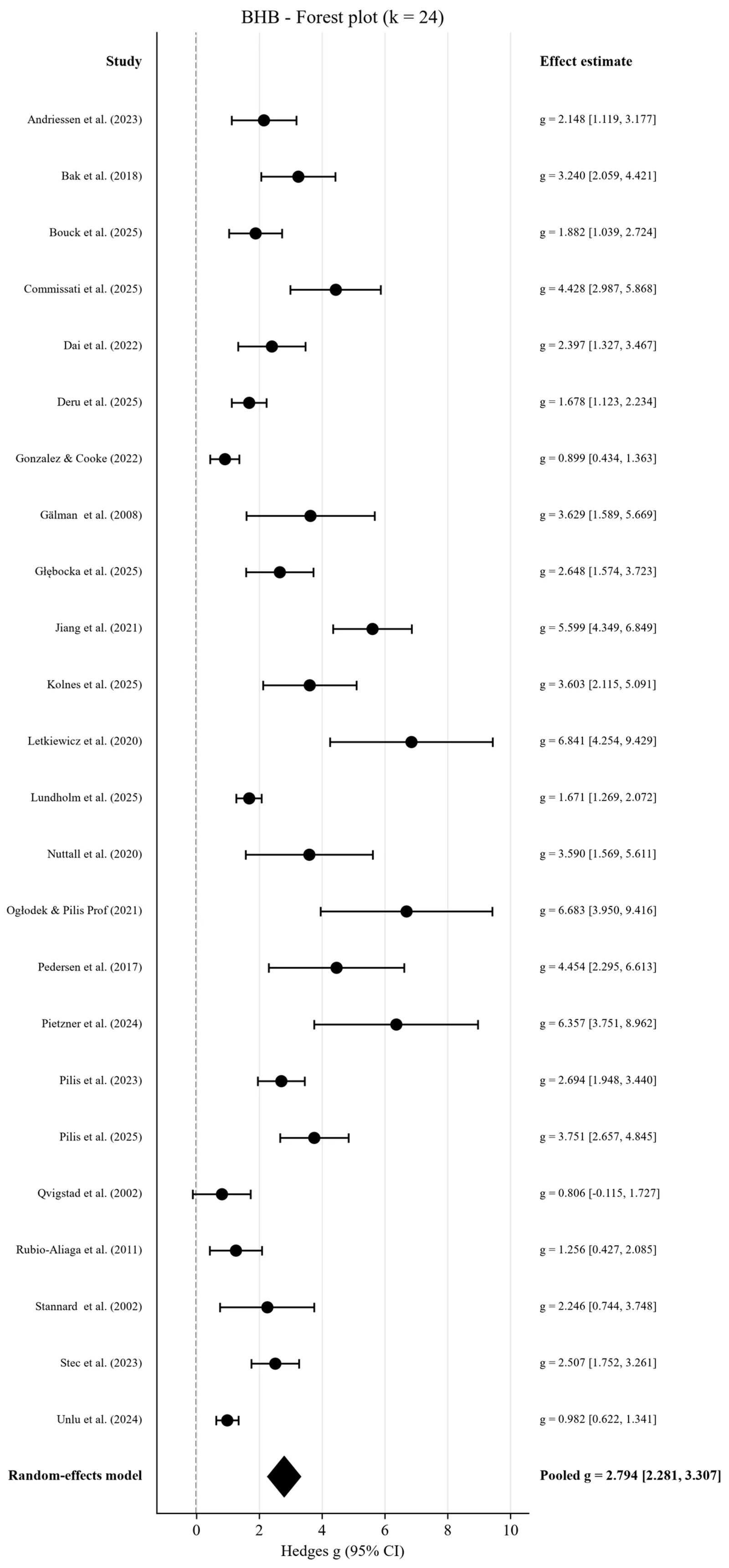
Forest plot of the effect of water-only fasting on circulating β-hydroxybutyrate (BHB) levels [6,10,12,14,15,22,23,24,25,27,40,41,42,43,44,45,46,47,48,49,51,52,53,54]. Circles indicate individual study effect estimates, while the rhombus represents the pooled effect estimate and its 95% confidence interval.

Twenty-four independent study-level estimates from 24 studies were included in the β-hydroxybutyrate analysis (Figure 3). The random-effects model demonstrated a large and statistically significant increase in circulating BHB concentrations following water-only fasting (Hedges’ g = 2.794, SE = 0.262; 95% CI: 2.281 to 3.307; *p* < 0.001). Between-study heterogeneity was substantial (Q = 176.37, *p* < 0.001; I^2^ = 87.0%; τ^2^ = 1.218), indicating considerable variability in the magnitude of BHB responses across studies.

##### Robustness and Sensitivity Analyses

The pooled effect remained statistically significant under Hartung–Knapp inference using the REML estimate of between-study variance (95% HK CI: 2.241 to 3.556). Estimates of between-study variance varied somewhat across alternative estimators but supported the same overall interpretation (τ^2^: DL = 1.218, PM = 2.186, REML = 2.068). The 95% prediction interval ranged from −0.237 to 5.825, indicating substantial between-study uncertainty and suggesting that an individual future study could observe a comparatively small or statistically non-significant effect despite the strongly positive pooled estimate.

Sensitivity analyses using alternative pre–post correlation assumptions produced highly similar pooled estimates (r = 0.3: g = 2.810, 95% CI: 2.282 to 3.338; r = 0.7: g = 2.772, 95% CI: 2.278 to 3.266). Restricting the analysis to the longest eligible fasting duration per study yielded an identical pooled estimate to the primary analysis (g = 2.794, 95% CI: 2.281 to 3.307; k = 24). In the overlap-adjusted analysis, which retained the largest sample within potentially overlapping cohorts, the pooled effect was slightly attenuated but remained large and statistically significant (g = 2.634, 95% CI: 2.128 to 3.140; k = 22). Thus, the direction and statistical significance of the pooled effect were unchanged across all sensitivity analyses.

##### Leave-One-Out Influence Analysis

Leave-one-out analyses confirmed that the pooled effect was not driven by any single study (Figure S3). Across 24 iterations, the pooled estimate ranged from g = 2.619 when Jiang et al. (2021) [49] was omitted to g = 2.908 when Gonzalez and Cooke (2022) [23] was omitted. All leave-one-out estimates remained statistically significant, with 95% confidence intervals excluding the null, and no individual study materially altered the direction or overall interpretation of the pooled effect.

##### Subgroup Analysis by Fasting Duration

**Figure 4:**
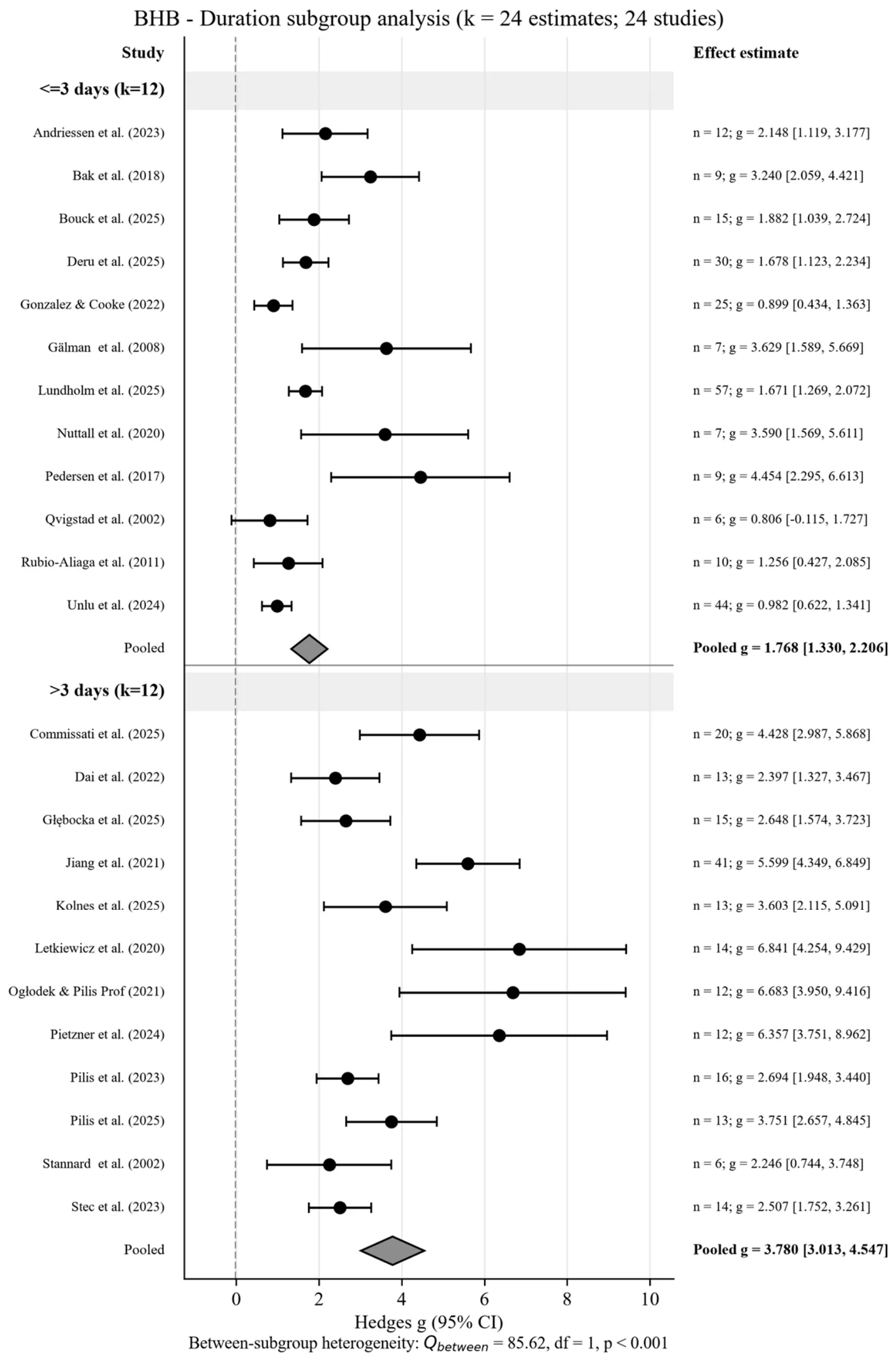
Forest plot of subgroup analysis by fasting duration (≤3 vs. >3 days) on circulating β-hydroxybutyrate [6,10,12,14,15,22,23,24,25,26,27,40,41,42,43,44,45,46,47,48,49,51,52,53,54]. Circles indicate individual study effect estimates, while the rhombus represents the pooled effect estimate and its 95% confidence interval.

Subgroup analysis showed a clear difference according to fasting duration (Figure 4). Fasting lasting ≤3 days was associated with a large and statistically significant increase in circulating BHB concentrations (k = 12; g = 1.768; 95% CI: 1.330 to 2.206; I^2^ = 74.7%). Fasting lasting >3 days produced a substantially larger pooled effect (k = 12; g = 3.780; 95% CI: 3.013 to 4.547; I^2^ = 76.7%). The between-subgroup difference was statistically significant (QBetween = 85.62, df = 1, *p* < 0.001), indicating that BHB elevations were markedly greater in fasting protocols extending beyond three days.

##### Duration Meta-Regression

**Figure 5:**
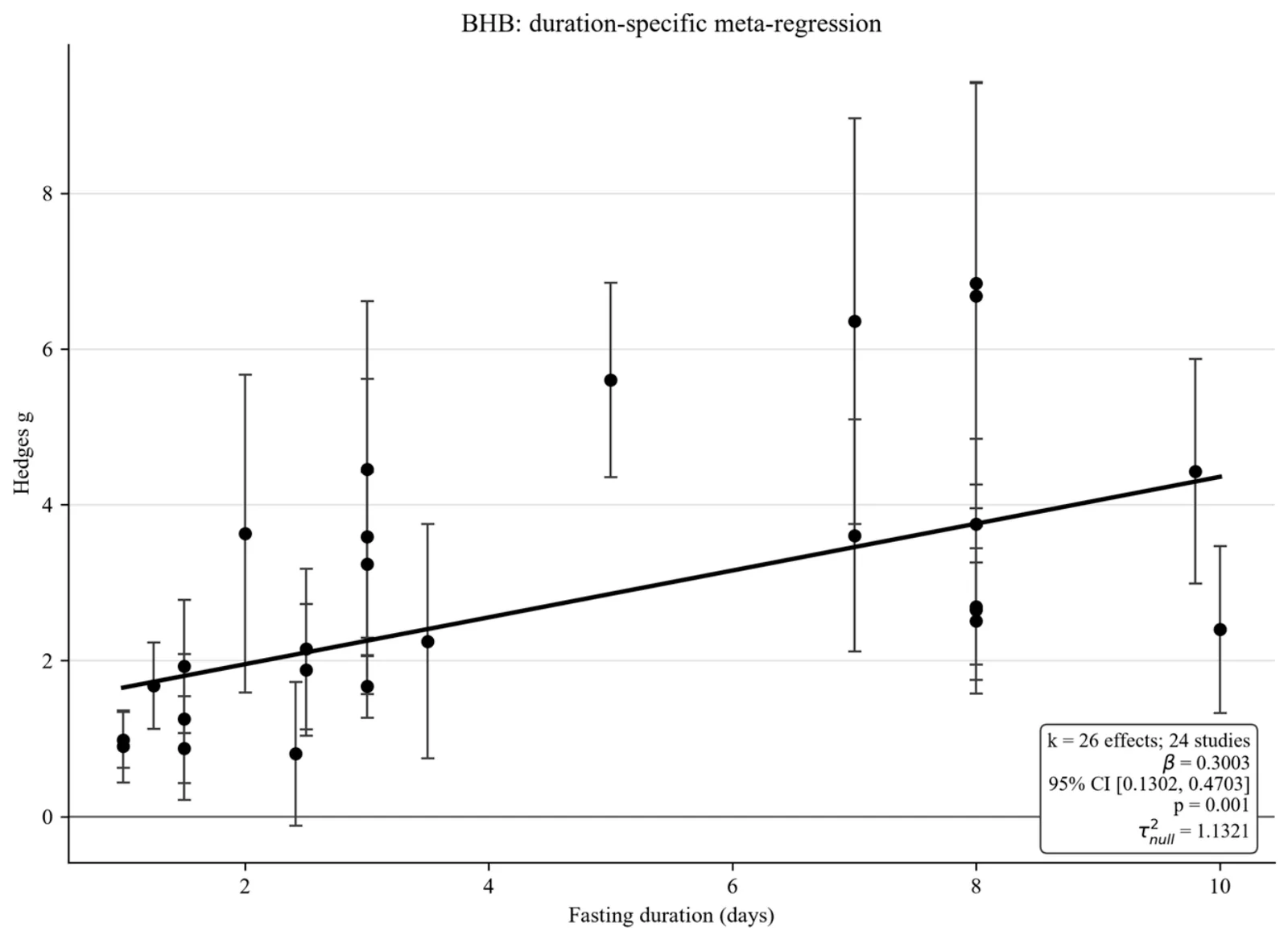
Meta-regression of fasting duration and β-hydroxybutyrate response.

Random-effects weighted least-squares meta-regression, based on 26 duration-specific estimates nested within 24 studies and using study-level cluster-robust standard errors, demonstrated a significant positive association between fasting duration and the BHB response (β = 0.300 per day, cluster-robust SE = 0.082; t = 3.66; 95% CI: 0.130 to 0.470; *p* = 0.001; Figure 5). Thus, each additional day of fasting was associated with an average increase of approximately 0.30 standardized units in the BHB effect size. Residual heterogeneity remained after accounting for fasting duration (QE = 27.69, df = 24), while fasting duration explained approximately 31% of the estimated between-study variance.

##### Publication Bias

Egger’s regression test indicated significant funnel-plot asymmetry consistent with small-study effects (intercept = 4.252, SE = 0.644; t = 6.60; df = 22; *p* < 0.001; Figure S4). This finding suggests that smaller studies tended to report larger BHB effect estimates. Accordingly, although the direction of the pooled effect was robust, its absolute magnitude may be overestimated in the available literature.

#### 3.2.3. Plasma Ketones

##### Overall Meta-Analysis

**Figure 6:**
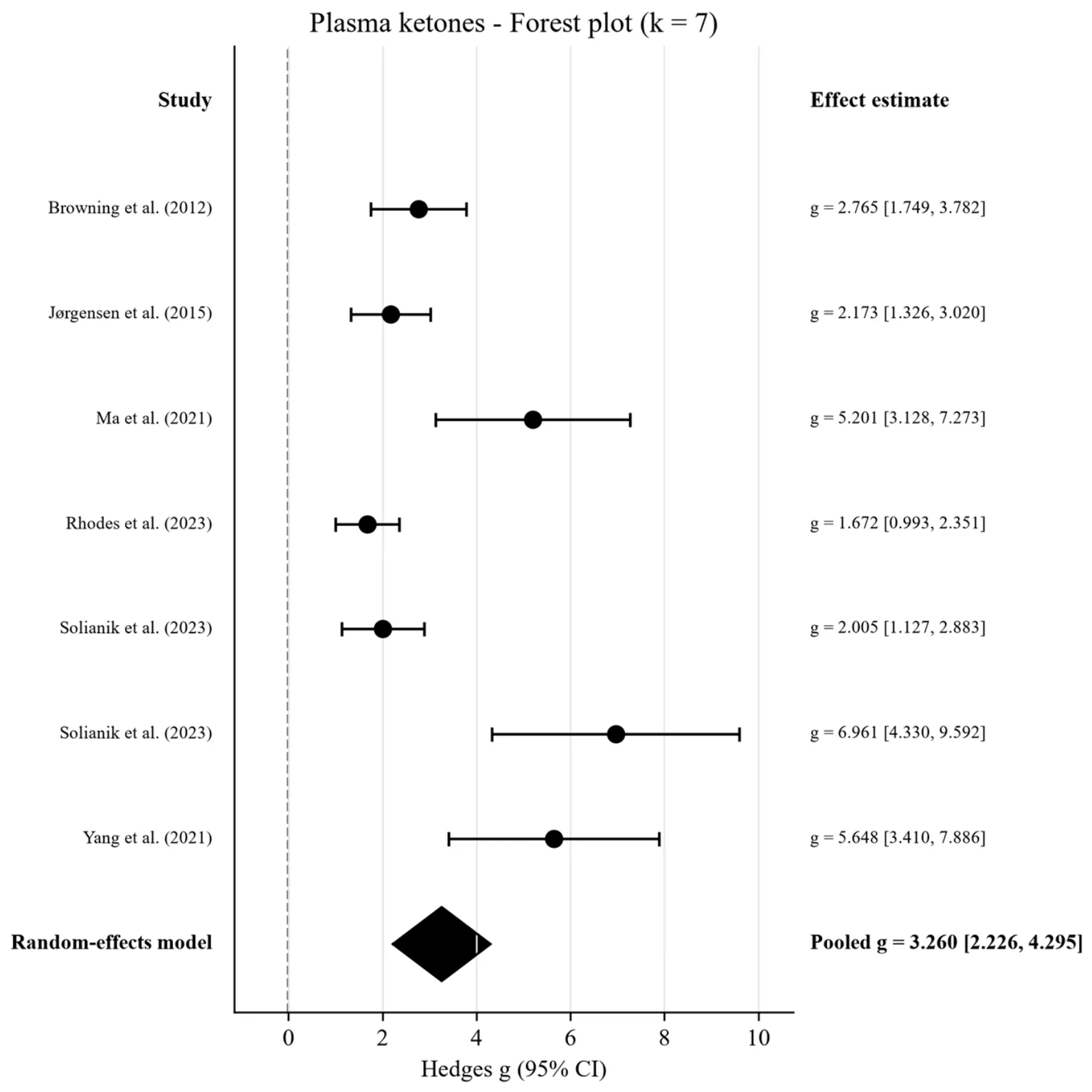
Forest plot of the effect of water-only fasting on plasma ketone concentrations [4,11,16,21,26,50]. Circles indicate individual study effect estimates, while the rhombus represents the pooled effect estimate and its 95% confidence interval.

Seven independent study-level estimates from six studies were included in the total plasma ketone analysis, with the independent 2-day and 6-day fasting arms of Solianik et al. (2023) [4] retained as separate estimates (Figure 6). The random-effects model demonstrated a large and statistically significant increase in circulating total plasma ketone concentrations following water-only fasting (Hedges’ g = 3.260, SE = 0.528; 95% CI: 2.226 to 4.295; *p* < 0.001). Between-study heterogeneity was substantial (Q = 32.62, *p* < 0.001; I^2^ = 81.6%; τ^2^ = 1.402), indicating considerable variability in the magnitude of plasma ketone responses across studies.

##### Robustness and Sensitivity Analyses

The pooled effect remained statistically significant under Hartung–Knapp inference using the REML estimate of between-study variance (95% HK CI: 1.819 to 5.142). Estimates of between-study variance differed substantially across alternative estimators (τ^2^: DL = 1.402, PM = 3.760, REML = 3.162), consistent with the pronounced heterogeneity and limited number of contributing studies. The 95% prediction interval ranged from −1.508 to 8.029, indicating substantial uncertainty in the effect expected in an individual future study and allowing for the possibility of a small or statistically non-significant response despite the strongly positive pooled estimate.

Sensitivity analyses using alternative pre–post correlation assumptions yielded very similar pooled estimates (r = 0.3: g = 3.280, 95% CI: 2.216 to 4.344; r = 0.7: g = 3.236, 95% CI: 2.234 to 4.238). Restricting the analysis to the longest eligible fasting duration per study yielded the same pooled estimate as the primary analysis (g = 3.260, 95% CI: 2.226 to 4.295). In the overlap-adjusted analysis, the pooled effect was somewhat attenuated but remained large and statistically significant (g = 2.911, 95% CI: 1.930 to 3.891; k = 6). The direction and statistical significance of the pooled effect were therefore unchanged across all sensitivity analyses.

##### Leave-One-Out Influence Analysis

Leave-one-out analyses showed that the pooled effect remained positive and statistically significant regardless of which study was omitted (Figure S5). Across the iterations, the pooled estimate ranged from g = 2.911 when Yang et al. (2021) [21] was omitted to g = 3.686 when Rhodes et al. (2023) [11] was omitted. Although the absolute magnitude of the pooled estimate showed some sensitivity to individual studies, no single study altered the direction or statistical significance of the overall effect.

##### Subgroup Analysis by Fasting Duration

**Figure 7:**
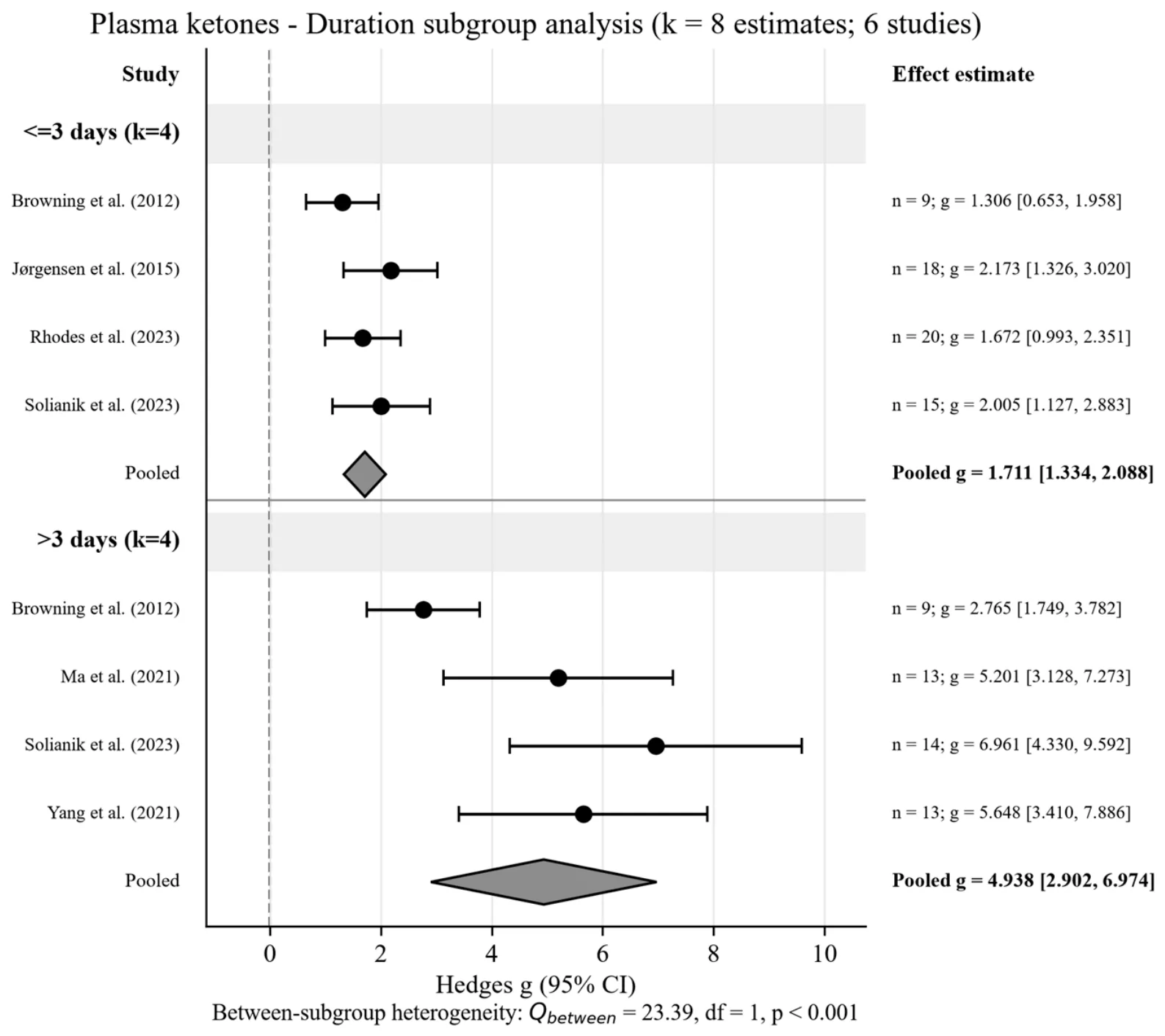
Forest plot of subgroup analysis by fasting duration (≤3 vs. >3 days) on plasma ketones [4,11,16,21,26,50]. Circles indicate individual study effect estimates, while the rhombus represents the pooled effect estimate and its 95% confidence interval.

Subgroup analysis suggested a pronounced difference according to fasting duration (Figure 7). Fasting lasting ≤3 days was associated with a large and statistically significant increase in total plasma ketone concentrations (k = 4; g = 1.711; 95% CI: 1.334 to 2.088; I^2^ = 2.3%). Fasting lasting >3 days was associated with a substantially larger pooled effect (k = 4; g = 4.938; 95% CI: 2.902 to 6.974; I^2^ = 78.3%). The between-subgroup difference was statistically significant (QBetween = 23.39, df = 1, *p* < 0.001).

However, this subgroup comparison should be interpreted as exploratory because Browning et al. (2012) [16] contributed repeated within-participant measurements at both 2 and 6 days, whereas Solianik et al. (2023) [4] contributed independent parallel fasting arms. In addition, the >3-day subgroup contained only a small number of estimates and showed substantial residual heterogeneity.

##### Duration Meta-Regression

**Figure 8:**
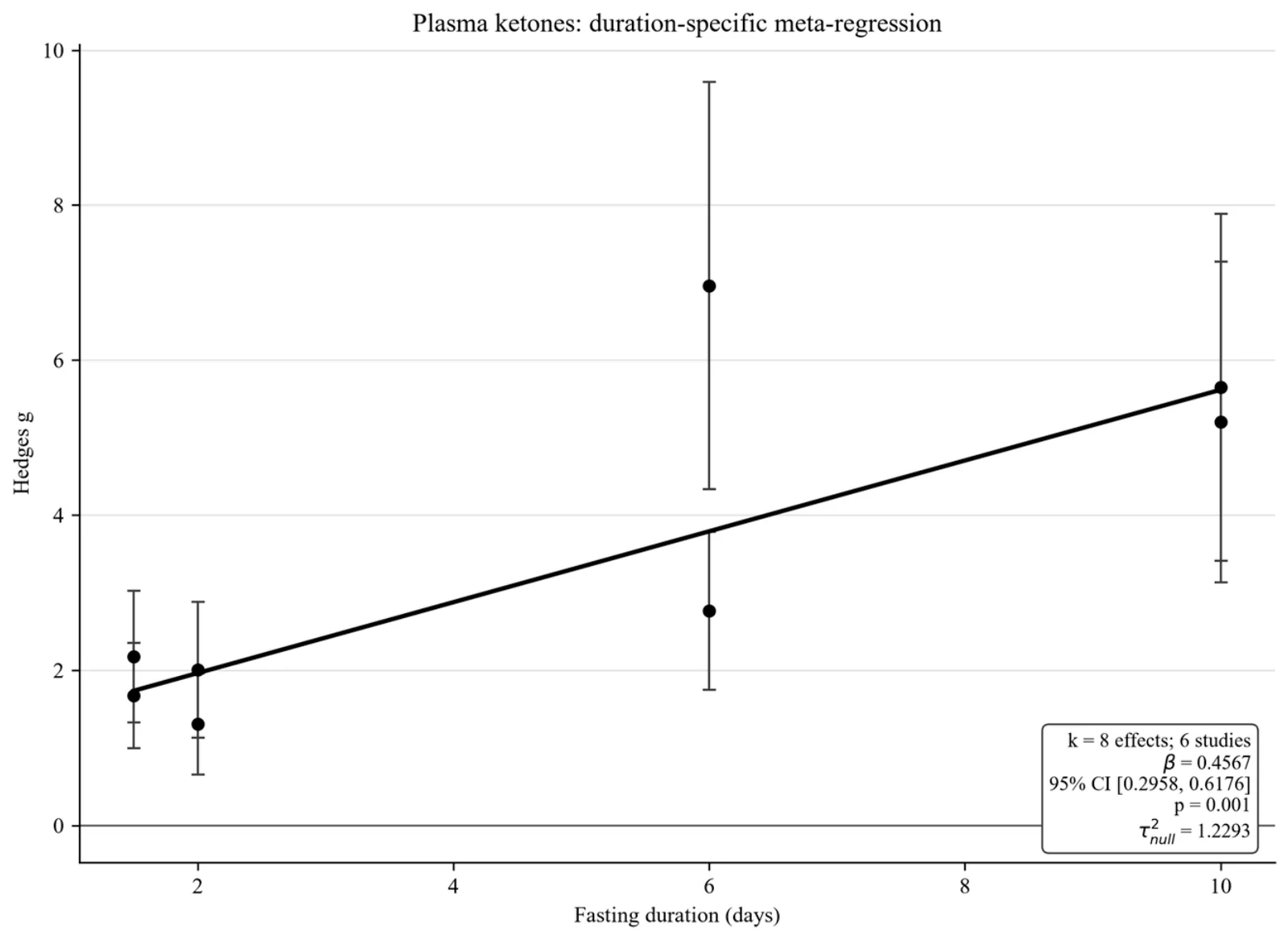
Meta-regression of fasting duration and plasma ketone response. Dots indicate individual study effect sizes, with error bars representing 95% confidence intervals. The solid line shows the meta-regression slope.

Random-effects weighted least-squares meta-regression, based on eight duration-specific estimates nested within six studies and using study-level cluster-robust standard errors, demonstrated a significant positive association between fasting duration and the total plasma ketone response (β = 0.457 per day, cluster-robust SE = 0.058; t = 7.88; 95% CI: 0.296 to 0.618; *p* = 0.001; Figure 8). Thus, each additional day of fasting was associated with an average increase of approximately 0.46 standardized units in the plasma ketone effect size. Residual heterogeneity was modest after accounting for fasting duration (QE = 4.55, df = 6), and fasting duration explained approximately 71% of the estimated between-study variance. Given that only six independent studies contributed to the analysis, these meta-regression findings should be considered exploratory.

##### Publication Bias

Egger’s regression test was not performed because fewer than 10 independent study-level estimates were available (k = 7; Figure S6). Visual inspection of the funnel plot suggested some asymmetry, with smaller studies tending to report larger effects. However, given the limited number of studies and the substantial between-study heterogeneity, this pattern should be interpreted as exploratory and may reflect small-study effects, genuine heterogeneity, or both.

## 4. Discussion

The present meta-analysis demonstrates that water-only fasting and other prolonged fasting protocols involving complete caloric abstinence are associated with large, statistically significant elevations in circulating ketone bodies in adults, with consistent increases across β-hydroxybutyrate, plasma ketones, and acetoacetate. Among these biomarkers, total plasma ketones exhibited the largest pooled standardized response, accompanied by substantial between-study heterogeneity. Importantly, both categorical subgroup analyses and continuous meta-regression revealed a clear duration-dependent pattern for β-hydroxybutyrate and total plasma ketones, indicating progressively greater ketone accumulation with longer fasting periods. Specifically, fasting durations exceeding three days were associated with markedly larger increases in β-hydroxybutyrate and plasma ketone concentrations compared with shorter protocols, while linear meta-regression confirmed a strong positive association between fasting duration and standardized ketone responses for both biomarkers. Sensitivity analyses across alternative correlation assumptions and between-study variance estimators supported the robustness of these findings, indicating that the overall findings were not materially influenced by analytical choices. The substantial heterogeneity observed likely reflects variability in fasting duration, participant characteristics (e.g., body composition, metabolic status, and sex), and experimental conditions (e.g., physical activity and measurement timing). Although fasting duration explained a significant proportion of this variability for β-hydroxybutyrate and total plasma ketones, other potential moderators could not be systematically examined due to inconsistent reporting across studies. Collectively, these results provide quantitative evidence that fasting-induced ketogenesis in humans follows a time-dependent trajectory, with prolonged fasting producing substantially greater elevations in circulating ketone bodies. Given the limited number of studies on plasma ketone outcomes, particularly in subgroup analyses, these findings should be interpreted as exploratory and warrant caution.

The observed duration-dependent increase in circulating ketone bodies is physiologically consistent with the progressive metabolic adaptations that occur during prolonged fasting [4,16,27,56]. Following the depletion of hepatic glycogen stores—typically within the first 24–48 h of food deprivation—there is a marked shift toward increased lipolysis and hepatic fatty acid oxidation, providing substrates for ketone body synthesis [5,19,20]. As fasting continues, mitochondrial β-oxidation in hepatocytes accelerates, leading to increased production of acetoacetate, which is subsequently reduced to β-hydroxybutyrate, the predominant circulating ketone due to its greater stability and transport efficiency [38,57]. Concurrently, peripheral tissues and the central nervous system increasingly rely on ketone bodies as oxidative fuels, facilitating glucose sparing and attenuating whole-body proteolysis [19,58]. These coordinated adaptations explain why ketone concentrations rise modestly during early fasting but increase substantially with longer fasting durations, as reflected in the larger effect sizes observed beyond three days for β-hydroxybutyrate and total plasma ketones in the present analysis. The pronounced increase in β-hydroxybutyrate observed during fasting further aligns with its central role in sustaining systemic energy demands during prolonged caloric deprivation [20,38,58].

The clear duration-dependent pattern identified in this meta-analysis for β-hydroxybutyrate and total plasma ketones may also help explain inconsistencies observed across individual fasting studies [1,17,45,58]. Short-term fasting protocols, often limited to 24–36 h, may capture only the initial phase of metabolic adaptation, during which ketone concentrations begin to rise but have not yet reached the higher levels characteristic of more prolonged fasting [4,16,27]. In contrast, studies implementing fasting durations beyond three days are more likely to reflect the later adaptive phase, marked by sustained hepatic ketogenesis and substantially elevated circulating ketone concentrations [40,41,53]. Without accounting for fasting duration, comparisons across studies may therefore obscure meaningful differences in metabolic magnitude, potentially contributing to variability in reported findings. The present synthesis indicates that fasting duration is not merely a methodological detail but an important moderator of β-hydroxybutyrate and total plasma ketone response magnitude. Accordingly, the interpretation of fasting-related metabolic outcomes should explicitly consider duration, particularly when comparing protocols that differ substantially in length.

Substantial between-study heterogeneity was observed for β-hydroxybutyrate and total plasma ketones, whereas heterogeneity for acetoacetate was low to moderate, reflecting the methodological and physiological diversity of the included studies. Fasting duration varied widely, and participant characteristics—including baseline metabolic status, body composition, and health profiles—were inconsistently reported, all of which may influence the magnitude of ketone responses [10,23,46]. In addition, most studies employed single-arm pre–post designs with relatively small sample sizes, increasing susceptibility to variability and imprecision [6,24,25,46].

Evidence of small-study effects was detected for β-hydroxybutyrate, while funnel-plot asymmetry for total plasma ketones was assessed visually only and should be considered exploratory given the limited number of independent estimates, suggesting that the magnitude of pooled effects may be overestimated, particularly for β-hydroxybutyrate. Nevertheless, the direction of effect was consistent across studies, and sensitivity analyses using alternative correlation assumptions and between-study variance estimators yielded comparable pooled estimates, supporting the robustness of the overall positive effects. These findings underscore the need for cautious interpretation of absolute effect magnitudes while reinforcing the reliability of the overall pattern of increased circulating ketone concentrations during fasting.

Using the NIH Quality Assessment Tool for Before–After (Pre–Post) Studies With No Control Group, 13 of the 30 included studies were rated as good and 17 as fair, with none rated as poor. The most frequent methodological limitations included small sample sizes, the absence of a concurrent non-fasting comparator, incomplete reporting of participant characteristics, reliance on graphically extracted data in several studies, and probable participant overlap among some prolonged-fasting cohorts. This methodological structure may increase susceptibility to bias, particularly in the estimation of effect magnitude. However, the consistency of effect direction across studies suggests that the observed increase in circulating ketone bodies is unlikely to be solely attributable to bias. Rather, these methodological limitations may have contributed to uncertainty or overestimation in the magnitude of the pooled effects. Therefore, the findings should be interpreted as robust in direction but with appropriate caution regarding their absolute magnitude. Because no study was rated as poor, a sensitivity analysis excluding studies on the basis of an unfavorable overall quality rating was not warranted.

Although fasting duration was identified as an important source of heterogeneity for β-hydroxybutyrate and total plasma ketones, other potentially relevant moderators—such as metabolic status, body composition, and study design—could not be formally examined. This was primarily due to inconsistent and incomplete reporting of these variables across the included studies, as well as limited sample sizes within subgroups. As a result, conducting additional moderator analyses would have yielded small, imbalanced subgroups and, consequently, unstable and potentially unreliable estimates. Future studies with more standardized reporting of participant and protocol characteristics are needed to better elucidate the contribution of these factors to variability in ketone responses.

Evidence of small-study effects was observed for β-hydroxybutyrate, whereas the apparent funnel-plot asymmetry for total plasma ketones was based on visual inspection only and should be interpreted as exploratory. This pattern suggests that the magnitude of the pooled β-hydroxybutyrate effect may be overestimated, while a similar possibility cannot be excluded for total plasma ketones. However, the consistency in the direction of effects across studies supports the robustness of the overall findings. Bias-adjustment methods such as trim-and-fill were considered; however, given the substantial between-study heterogeneity and the relatively small number of studies for certain outcomes, these methods were deemed potentially unstable and prone to generating misleading corrections. Accordingly, we chose to interpret the results conservatively and have explicitly emphasized that the reported effect sizes, particularly for β-hydroxybutyrate, may overestimate the underlying effect magnitude.

Inspection of studies contributing the largest standardized effects indicates that these were predominantly derived from prolonged fasting protocols conducted in metabolically healthy participants under strictly supervised water-only conditions. For example, Ogłodek and Pilis (2021) [40] and Letkiewicz et al. (2020) [41] both implemented 8-day fasting interventions in healthy middle-aged men and reported marked elevations in circulating β-hydroxybutyrate, despite modest sample sizes. Similarly, Pietzner et al. (2024) [53] observed pronounced ketone responses following a 7-day fasting protocol in carefully screened healthy adults. Comparable patterns were reported in longer 10-day fasting studies by Ma et al. (2021) [26] and Yang et al. (2021) [21], where substantial ketone accumulation occurred in healthy male cohorts. In contrast, studies employing shorter fasting durations or less standardized designs tended to yield smaller effects. Although these comparisons are descriptive, they suggest that prolonged fasting exposure, protocol rigor, and participant selection may jointly contribute to the large effect sizes observed in certain investigations and partially explain the substantial heterogeneity observed for β-hydroxybutyrate and total plasma ketone outcomes.

Several methodological strengths enhance the interpretability of the present findings. To our knowledge, this study provides the first quantitative synthesis specifically examining the duration-dependent effects of water-only fasting and other prolonged fasting protocols involving complete caloric abstinence on circulating ketone biomarkers in humans. Separate analyses were conducted for β-hydroxybutyrate, plasma ketones, and acetoacetate, allowing biomarker-specific characterization of fasting responses. The use of random-effects models, complemented by multiple between-study variance estimators and sensitivity analyses across alternative pre–post correlation assumptions, further strengthened the robustness of the pooled estimates. In addition, both categorical subgroup analyses and continuous meta-regression were applied to evaluate the influence of fasting duration, providing convergent evidence for a time-dependent trajectory of β-hydroxybutyrate and total plasma ketone accumulation, while the corresponding evidence for acetoacetate remained limited. Collectively, these analytical strategies reduce reliance on single-model assumptions and offer a comprehensive framework for quantifying fasting-induced ketogenesis across heterogeneous study designs.

Several limitations should be acknowledged. Most included studies employed single-arm pre–post designs and relatively small sample sizes, limiting causal inference and increasing susceptibility to random error. Fasting protocols varied substantially in duration and implementation, including differences in supervision, hydration practices, and experimental conditions, and participant characteristics such as baseline metabolic status and body composition were inconsistently reported, restricting more granular moderator analyses. In addition, the number of available studies on acetoacetate was limited compared with those on β-hydroxybutyrate and plasma ketones, reducing precision for this biomarker. Although sensitivity analyses supported the stability of pooled estimates, formal evidence of small-study effects for β-hydroxybutyrate, together with exploratory funnel-plot asymmetry for total plasma ketones, indicates that absolute effect magnitudes should be interpreted cautiously. The limited number of studies contributing to the acetoacetate and total plasma ketone analyses also reduced the precision of subgroup and meta-regression estimates, particularly for duration-dependent effects. Future research would benefit from larger, controlled trials with standardized fasting protocols and serial ketone measurements across multiple time points to more precisely characterize the temporal dynamics of fasting-induced ketogenesis. Such studies would further refine the understanding of duration-specific metabolic responses and improve comparability across fasting interventions.

## 5. Conclusions

This systematic review and meta-analysis demonstrates that water-only fasting and other prolonged fasting protocols involving complete caloric abstinence are associated with large and statistically significant increases in circulating ketone bodies in adults, with consistent elevations observed across β-hydroxybutyrate, total plasma ketones, and acetoacetate. Among the included studies explicitly implementing water-only fasting, fasting durations ranged from 24 h to approximately 10 days. Among these biomarkers, total plasma ketones exhibited the largest pooled standardized response, accompanied by substantial between-study heterogeneity. Both categorical subgroup analyses and continuous meta-regression revealed a clear duration-dependent pattern for β-hydroxybutyrate and total plasma ketones, indicating progressively greater ketone elevations with longer fasting periods. Specifically, fasting durations exceeding three days were associated with markedly larger increases in β-hydroxybutyrate and total plasma ketone concentrations compared with shorter fasting protocols, and linear meta-regression confirmed a strong positive association between fasting duration and both biomarker responses. Sensitivity analyses across alternative pre–post correlation assumptions and between-study variance estimators supported the robustness of pooled estimates. However, formal evidence of small-study effects for β-hydroxybutyrate, together with exploratory funnel-plot asymmetry for total plasma ketones, suggests that the magnitude of some pooled effects may be overestimated, underscoring the need for cautious interpretation. Collectively, these findings provide quantitative evidence that fasting-induced ketogenesis in humans follows a time-dependent trajectory, particularly for β-hydroxybutyrate and total plasma ketones, with prolonged fasting producing substantially greater ketone accumulation. From a clinical perspective, these results provide a quantitative reference for the expected magnitude and timing of ketone responses during fasting, which may inform the design and monitoring of fasting-based or ketogenic interventions. However, given the predominance of non-controlled study designs, these findings should be interpreted as physiological benchmarks rather than direct evidence of clinical efficacy.

## Figures and Tables

**Table 1:** Characteristics of Included Studies and Fasting Protocols [4,6,10,11,12,14,15,16,21,22,23,24,25,26,27,40,41,42,43,44,45,46,47,48,49,50,51,52,53,54].

Author (Year), Country	Study Design & Population	Fasting Protocol & Duration	Reported Outcomes
Andriessen et al. (2023), Netherlands [27]	Randomized cross-over trial conducted in healthy, young, lean male volunteers (*n* = 12).	Prolonged fasting for up to 60 h (assessments at 36 and 60 h).	Plasma β-hydroxybutyrate (mmol/L) concentrations increased from 0.034 ± 0.014 to 0.619 ± 0.186 at 36 h and from 0.034 ± 0.014 to 2.236 ± 0.282 at 60 h (mean ± SD).
Bak et al. (2018), Denmark [22]	Randomized crossover study conducted in 18 apparently healthy young men aged 20–35 years, including nine lean participants (BMI 19–23 kg/m^2^) and nine obese participants (BMI 32–40 kg/m^2^).	Prolonged fasting for 72 h.	Plasma β-hydroxybutyrate concentrations (mM) increased from 0.3 ± 0.1 to 3.0 ± 0.6 in lean participants (n = 9) and from 0.3 ± 0.2 to 1.9 ± 0.6 in obese participants (n = 9) (mean ± SD).
Browning et al. (2012), USA [16]	Prospective single-arm interventional metabolic study in healthy individuals (total sample *n* = 18; 9 males, 9 females). Women were pre-menopausal. Reported ages: women 24 (23–42) years, men 21 (21–23) years. This subgroup includes women only (*n* = 9).	Prolonged fasting for 48 h.	Plasma ketone concentrations (mmol/L) increased in women from 0.05 ± 0.035 to 0.33 ± 0.31 after 2 days and from 0.05 ± 0.035 to 1.22 ± 0.39 after 6 days; in men, values increased from 0.09 ± 0.035 to 0.41 ± 0.14 after 2 days and from 0.09 ± 0.035 to 1.94 ± 0.62 after 6 days (mean ± SD)
Bouck et al. (2025), Canada [45]	Randomized crossover trial in 15 healthy adults (6 F, 9 M; mean age 26.5 ± 4.3 y; VO_2_peak 51 ± 8 mL/kg/min); no-exercise (FAST) arm used.	60-h water-only fast (non-caloric black coffee/tea permitted); blood sampled at 12, 36, and 60 h; continuous glucose monitoring for safety.	Serum β-hydroxybutyrate (mmol/L) increased from ≈0.08 ± 0.01 (12 h) to ≈2.1 ± 1.02 at 60 h (also ≈0.62 ± 0.27 at 36 h); mean ± SD, values digitized from Figure 1.
Commissati et al. (2025), Australia [46]	Prospective single-arm interventional experimental human study conducted in middle-aged volunteers (*n* = 20; 9 males, 11 females) with a mean age of 52.2 ± 11.8 years and a mean body mass index of 28.8 ± 6.4 kg/m^2^.	Medically supervised water-only fasting for approximately 10 days (mean duration 9.8 ± 3.1 days).	Plasma β-hydroxybutyrate concentrations increased from 0.6 ± 0.9 to 5.0 ± 1.0 mmol/L (mean ± SD).
Dai et al. (2022), China [14]	Prospective single-arm experimental human study conducted in male participants (*n* = 13) with a mean age of 39.6 ± 7.9 years (range 28–55), mean body weight of 72.1 ± 11.9 kg (range 54.2–92.2), and mean body mass index of 24.6 ± 3.5 (range 19.2–31.9).	Water-only fasting for 10 consecutive days under medical supervision.	Plasma β-hydroxybutyrate concentrations increased from 0.18 ± 0.04 to 4.15 ± 1.57 mmol/L (mean ± SD).
Deru et al. (2025), USA [47]	Randomized crossover study conducted in healthy adults (*n* = 30; 16 males, 14 females) aged 18 years and older who had been weight stable (±3% body weight) for the past 3 months.	Prolonged fasting for 30 h.	Plasma β-hydroxybutyrate concentrations increased from 0.145 ± 0.071 to 1.067 ± 0.567 mM (mean ± SD).
Gälman et al. (2008), Sweden [48]	Prospective, multi-cohort, observational and interventional human metabolic study including healthy volunteers (*n* = 7; 3 males, 4 females) and female patients with rheumatoid arthritis (*n* = 5).	Water-only fasting for 48 h (healthy cohort) and 7 days (rheumatoid arthritis cohort).	Serum 3-hydroxybutyrate increased from ≈0.08 to ≈3.24 mmol/L after 48 h of fasting in healthy volunteers (n = 7; mean ± SEM; data originally from Arner et al. 2003 [55]). The 7-day arm in rheumatoid-arthritis patients was excluded per the disease-population criterion.
Głębocka et al. (2025), Poland [15]	Prospective, single-arm, pre–post interventional study conducted in 15 male participants who had previously completed fasting sessions of at least 7 days, during which they consumed only mineral water.	Water-only fasting for 8 days (mineral water only).	Plasma β-hydroxybutyrate concentrations increased from 0.142 ± 0.13 to 4.537 ± 1.63 mmol/L (mean ± SD).
Gonzalez & Cooke (2022), USA [23]	Randomized controlled crossover study conducted in healthy young adults (*n* = 25; 14 males, 11 females) with a mean age of 23 ± 3 years, height 176 ± 16 cm, body mass 76 ± 16 kg, body mass index 24 ± 4 kg/m^2^, and body surface area 1.9 ± 0.2 m^2^.	Prolonged fasting for 24 h.	Plasma β-hydroxybutyrate concentrations increased from 0.128 ± 0.13 to 0.536 ± 0.49 mmol/L (mean ± SD).
Jiang et al. (2021), China [49]	Prospective single-arm clinical trial conducted in healthy, normal-weight adults (*n* = 41; 17 males, 24 females).	Water-only fasting for 5 days.	Serum acetoacetic acid concentrations increased from 405.80 ± 115.94 to 1797.10 ± 86.96 µg/mL, while serum 3-hydroxybutyric acid concentrations increased from 45.39 ± 13.82 to 2373.68 ± 414.74 µg/mL (mean ± SD).
Jørgensen et al. (2015), Denmark [50]	Prospective clinical study conducted in young healthy white men (total sample *n* = 39; 21 SGA and 18 AGA). Ketone analysis was available for a subgroup of 18 participants.	Prolonged fasting for 36 h (water allowed ad libitum).	Blood ketone concentrations increased from 0.08 ± 0.02 to 2.21 ± 0.23 mmol/L (mean ± SE).
Kolnes et al. (2025), Norway [10]	Prospective single-arm interventional study with a pre–post design conducted in young, healthy participants (*n* = 13; 7 males, 6 females) without significant medical history and not using prescription drugs or tobacco products.	Prolonged fasting for 7 days.	Plasma β-hydroxybutyrate concentrations increased from 0.07 ± 0.10 to 4.01 ± 1.07 mM (mean ± SD).
Letkiewicz et al. (2020), Poland [41]	Prospective single-arm pre–post interventional human study conducted in healthy men (*n* = 14), aged 35–60 years (mean age 49.64 ± 9.30 years; median 52.00).	Water-only fasting for 8 days.	Plasma β-hydroxybutyrate concentrations increased from 0.29 ± 0.20 to 4.69 ± 0.68 mmol/L (mean ± SD).
Lundholm et al. (2025), USA [51]	Quality improvement report including adult participants (total sample *n* = 64; 14 males, 50 females), with ketone data available for 57 individuals. Participants had a mean age of 48 ± 17 years and mean BMI of 30 ± 7 kg/m^2^. Diabetes was present in 12 (19%) patients, and 14 (22%) had a history of bariatric surgery.	Prolonged fasting for up to 72 h.	Plasma β-hydroxybutyrate concentrations increased from a median of 0.57 (IQR: 0.31–0.91) to 2.73 (IQR: 1.75–3.59) mmol/L.
Ma et al. (2021), China [26]	Prospective controlled interventional repeated-measures study conducted in healthy male volunteers (total *n* = 13; Newbie group = 6, Experienced group = 7), aged between 28 and 55 years (mean age 40 ± 8 years).	Water-only fasting for 10 consecutive days.	Ketone body concentrations increased from 0.18 ± 0.04 to 5.87 ± 0.99 mmol/L in the experienced group (n=7) and from 0.17 ± 0.05 to 5.4 ± 0.85 mmol/L in the fasting-naïve group (n=6) (mean ± SD).
Nuttall et al. (2020), USA [24]	Randomized crossover study conducted in males with type 2 diabetes mellitus (*n* = 7).	Prolonged fasting for 72 h.	β-Hydroxybutyrate increased from 0.091 ± 0.210 mmol/L at baseline to 2.233 ± 0.529 mmol/L after 72 h of fasting (mean ± SD).
Ogłodek & Pilis (2021), Poland [40]	Prospective single-arm pre–post experimental human study conducted in healthy male volunteers (*n* = 12) with a mean age of 50.25 ± 14.18 years.	Water-only fasting for 8 days.	Plasma β-hydroxybutyrate concentrations increased from 0.30 ± 0.21 to 4.77 ± 0.70 mmol/L (mean ± SD).
Pedersen et al. (2017), Denmark [52]	Randomized, placebo-controlled crossover study conducted in healthy overweight and obese men (*n* = 9).	Prolonged fasting for 72 h.	Plasma β-hydroxybutyrate concentrations increased from a geometric mean of 15.8 (95% CI: 11.4–20.3) to 2327 (95% CI: 1598–3056) μmol/L.
Pietzner et al. (2024), Germany [53]	Prospective longitudinal interventional study conducted in apparently healthy non-smoking participants (*n* = 12; 7 males, 5 females), primarily of white-European ancestry. Exclusion criteria included any known disease and body fat percentage below 15% for females and 12% for males.	Water-only fasting for 7 days.	Plasma β-hydroxybutyrate concentrations increased from 0.19 ± 0.27 to 4.18 ± 0.67 mM (mean ± SD).
Pilis et al. (2023), Poland [42]	Prospective, single-arm, within-subject experimental intervention study conducted in apparently healthy male volunteers (*n* = 16), aged 53.38 ± 13.73 years.	Water-only fasting for 8 days.	Plasma β-hydroxybutyrate concentrations increased from 0.30 ± 0.20 to 4.30 ± 1.42 mmol/L under resting conditions and from 0.21 ± 0.115 to 3.68 ± 1.34 mmol/L under exercise conditions (mean ± SD).
Pilis et al. (2025), Poland [43]	Prospective, single-group experimental intervention study conducted in healthy, middle-aged men (*n* = 13; mean age 57.25 ± 9.93 years; body mass 81.72 ± 9.19 kg; height 178.54 ± 4.77 cm).	Water-only fasting for 8 days.	Plasma β-hydroxybutyrate concentrations increased from 0.31 ± 0.21 to 4.55 ± 1.04 mmol/L under resting conditions and from 0.21 ± 0.16 to 3.86 ± 1.06 mmol/L under exercise conditions (mean ± SD).
Qvigstad et al. (2002), Norway [6]	Controlled crossover experimental study conducted in healthy, non-diabetic subjects (total sample *n* = 14; 9 males, 5 females), aged 18–25 years, with a mean body mass index of 23.2 ± 0.8 kg/m^2^. Beta-hydroxybutyrate analysis was available for a subgroup of six participants.	Prolonged fasting for 58 h.	Plasma β-hydroxybutyrate concentrations increased from 0.01 ± 0.02 to 1.84 ± 0.79 mmol/L (mean ± SE).
Rhodes et al. (2023), USA [11]	Rigorously controlled pilot clinical study conducted in young healthy participants (*n* = 20; 10 males, 10 females; mean age 27.5 ± 4.35 years).	Prolonged fasting for 36 h.	Plasma acetoacetate concentrations increased from 0.75 ± 0.86 to 9.89 ± 5.37 mg/100 ml (mean ± SD). Circulating ketone body concentrations increased from 191.6 ± 129.9 to 2284 ± 1261 μmol/L (mean ± SD).
Rubio-Aliaga et al. (2011), Scotland [12]	Single-arm, repeated-measures experimental study conducted in a small and heterogeneous group of human volunteers (*n* = 10; 3 males, 7 females) covering a wide range of BMI (18.5–39.7 kg/m^2^), ages (25–56 years), and fasting glucose levels (86–119 mg/dL).	Prolonged fasting for 36 h.	Plasma acetoacetate concentrations increased from 0.01 ± 0.01 to 0.12 ± 0.09 mM (mean ± SD). Plasma 3-hydroxybutyrate concentrations increased from 0.07 ± 0.04 to 1.17 ± 0.82 mM (mean ± SD).
Solianik et al. (2023), Lithuania [4]	Single-centre, parallel-group, single-blind randomized controlled trial including healthy adult males with BMI between 19.5 and 29.9 kg/m^2^ (total sample *n* = 43; 2-day fasting *n* = 15, 6-day fasting *n* = 14, control *n* = 14). Participant characteristics were as follows: 2-day fast group (age 27.2 ± 8.8 years; weight 79.7 ± 11.4 kg; BMI 24.2 ± 3.2 kg/m^2^), 6-day fast group (age 31.0 ± 6.2 years; weight 84.7 ± 10.0 kg; BMI 24.7 ± 1.9 kg/m^2^), and control group (age 27.4 ± 7.7 years; weight 77.8 ± 13.2 kg; BMI 23.7 ± 2.5 kg/m^2^).	Prolonged fasting for 2 days and 6 days (parallel non-fasting control).	Capillary blood ketone concentrations (mmol/L) increased from 0.176 ± 0.11 to 1.79 ± 0.81 in the 2-day fasting group (n = 15) and from 0.21 ± 0.20 to 5.40 ± 0.78 in the 6-day fasting group (n = 14) (mean ± SD). The parallel non-fasting control group was not included in the meta-analysis.
Stannard et al. (2002), Australia [25]	Prospective single-group, pre–post experimental human study conducted in healthy, nondiabetic, physically fit males (*n* = 6), with a mean age of 35 years (range 23–55 years).	Water-only fasting for 84 h.	Whole blood 3-hydroxybutyrate concentrations increased from 0.05 ± 0.03 to 1.53 ± 0.24 mmol/L (mean ± SD).
Stec et al. (2023), Poland [44]	Prospective interventional study conducted in fourteen apparently healthy men aged 35–60 years, including younger participants (*n* = 6, age 38.5 ± 9.09 years) and older participants (*n* = 8, age 54.00 ± 5.55 years).	Water-only fasting for 8 days.	Plasma β-hydroxybutyrate concentrations increased from 0.317 ± 0.20 to 4.25 ± 1.50 mmol/L under resting conditions and from 0.21 ± 0.16 to 3.60 ± 1.40 mmol/L under exercise conditions (mean ± SD).
Unlu et al. (2024), USA [54]	Randomized crossover clinical study conducted in 44 participants (30 males, 14 females) with a wide range of body mass index values (17.6–48.6 kg/m^2^; mean BMI 31.7 ± 8.3 kg/m^2^).	Water-only fasting for 24 h.	Plasma β-hydroxybutyrate concentrations increased from 0.9 ± 0.2 to 5.2 ± 4.4 mg/dL (mean ± SD).
Yang et al. (2021), China [21]	Prospective within-subject experimental human study conducted in healthy male volunteers (*n* = 13), aged 28–55 years (mean age 39.62 ± 8.13 years).	Water-only fasting for 10 consecutive days.	Circulating ketone body concentrations increased from 0.2 ± 0.0 to 5.6 ± 0.9 mmol/L (mean ± SD).

## Data Availability

The original contributions presented in this study are included in the article/Supplementary Materials. Further inquiries can be directed to the corresponding author.

## Supplementary Materials

The following supporting information can be downloaded at: https://www.mdpi.com/article/10.3390/ijms27219570/s1.

## Abbreviations

The following abbreviations are used in this manuscript:

BHB	β-hydroxybutyrate
WoS	Web of Science
SE	Standard Error
SD	Standard Deviation
IQR	Interquartile Range
